# Network for forest by-products charcoal, resin, tar, potash (COST Action EU-PoTaRCh)

**DOI:** 10.12688/openreseurope.18160.1

**Published:** 2024-08-13

**Authors:** Magdalena Zborowska, Jakub Brózdowski, Jakob Starlander, Jiri Woitsch, Erika Ribechini, Rodica-Mariana Ion, Oliver Nelle, Koen Deforce, Anna Varga, Péter Szabó, Elena Badea, Johannes Tintiner-Olifiers, Katja Tikka, Jeannette Jacqueline Lucejko

**Affiliations:** 1 Faculty of Forestry and Wood Technology, Poznań University of Life Sciences, Poznań, 60-637, Poland; 2 Universität Bern, Bern, 3012, Switzerland; 3 Institute of Ethnology of the Czech Academy of Sciences, Praha, 11000, Czech Republic; 4 Department of Chemistry and Industrial Chemistry, University of Pisa, Pisa, 56124, Italy; 5 National Institute of Research and Development for Chemistry and Petrochemistry - ICECHIM, Bucharest, 060021, Romania; 6 Landesamt für Denkmalpflege im Regierungspräsidium Stuttgart, Gaienhofen-Hemmenhofen, 78343, Germany; 7 Ghent University, Gent, 9000, Belgium; 8 Royal Belgian Institute of Natural Sciences, Brussels, 1000, Belgium; 9 Padon Foundation, Budapest, 1088, Hungary; 10 Institute of Botany of the Czech Academy of Sciences, Pruhonice, 25243, Czech Republic; 11 Department of Environmental Studies, Masaryk University, Faculty of Social Studies, Brno, 60200, Czech Republic; 12 University of Craiova, Craiova, 200585, Romania; 13 Denkstatt - denkstatt GmbH, Wien, 1140, Austria; 14 University of Helsinki, Helsinki, 00014, Finland

**Keywords:** Cultural heritage, history, archaeology, forest by-products, bio-economy, chemical compostion, physical properties

## Abstract

COST Action EU-PoTaRCh establishes a network focused on the past, present and future use of the major forest by-products in Europe. The Action emphasizes forest by-products, mainly potash, tar, resin, and charcoal (PoTaRCh), as representatives of the traditional heritage of forest exploitation, including extractives used along the time for their specific chemical, biological activity and therapeutic effect. The scientific objective of the Action is to demonstrate the value of these products for the communities and their socio-economic growth, as well as their impact on biodiversity and climate throughout time. The general aim is to identify and assess changes in production and use of PoTaRCh and extractives, along with their social and environmental impacts on sustainable development, and draw lessons for the future based on this legacy. In this way, the Action supports the sustainable use of forests and the transfer of knowledge about circular economy practices, as a contribution to the systemic shift to the new circular bio-economy.

A key component of the Action is to support stakeholders that are knowledgeable about PoTaRCh goods and are interested in them because they are used in production, education and promotion of our natural and cultural heritage. The Action meets a wide range of demands thanks to the participation of partners with various activity profiles (museums, state forests, forest industry, tradition bearers associations, tourism industry, etc.). The EU-PoTaRCh Action highlights three COST inclusivity policies: the participation of inclusivity Target Countries with a long history of developing PoTaRCh, a special emphasis on gender balance, and the involvement of young researchers in leadership positions.

## Plain Language Summary

COST Action EU-PoTaRCh has established a network dedicated to exploring the past, present, and future use of major forest by-products in Europe. This initiative highlights key forest by-products, specifically potash, tar, resin, and charcoal (PoTaRCh), as important elements of traditional forest exploitation heritage. These products have been valued over time for their unique chemical and biological properties, as well as their therapeutic effects.

The scientific goal of the Action is to demonstrate the value of these products to communities, showcasing their role in socio-economic growth and their impact on biodiversity and climate over the ages. The general aim is to identify and assess changes in the production and use of PoTaRCh and other extractives, along with their social and environmental impacts on sustainable development. By understanding this legacy, we can draw lessons for the future.

In doing so, the Action supports the sustainable use of forests and promotes knowledge about circular economy practices, contributing to the systemic shift towards a new circular bio-economy.

A key component of the Action is supporting stakeholders who are knowledgeable about PoTaRCh goods and interested in their use for production, education, and the promotion of our natural and cultural heritage. The Action meets a wide range of demands by involving partners with diverse activity profiles, including museums, state forests, the forest industry, tradition bearers' associations, and the tourism industry.

The EU-PoTaRCh Action emphasizes three COST inclusivity policies: the participation of Inclusivity Target Countries with a rich history of developing PoTaRCh, a special focus on gender balance, and the involvement of young researchers in leadership positions.

## EU-PoTaRCh: Celebrating and Innovating Forest By-Products

COST Action EU-PoTaRCh weaves a dynamic network focused on the timeless and evolving uses of Europe’s key forest by-products: potash, tar, resin, and charcoal (PoTaRCh). These natural treasures, rooted in traditional forest practices, are celebrated for their unique chemical, biological, and therapeutic properties.

The initiative’s scientific mission is to showcase how these by-products have historically fueled community growth, enhanced economic vitality, and impacted biodiversity and climate. By tracing their journey from past to present, EU-PoTaRCh aims to understand their social and environmental roles, promoting sustainable development and learning valuable lessons for the future. This endeavor champions the principles of a circular bio-economy, where resources are reused and recycled.

Central to EU-PoTaRCh is the support for stakeholders who are passionate about PoTaRCh products and their cultural heritage. These include experts involved in production, education, and promotion. The Action brings together a rich tapestry of partners—museums, state forests, the forest industry, tradition bearers, and the tourism sector—to meet diverse needs and foster collaboration.

The initiative also prioritizes inclusivity by engaging countries with a rich history of PoTaRCh development, ensuring gender balance, and promoting young researchers in leadership roles. By embracing these principles, EU-PoTaRCh not only preserves the legacy of forest by-products but also drives innovation and sustainability, enhancing cultural and economic resilience across Europe.

**Figure 1.**
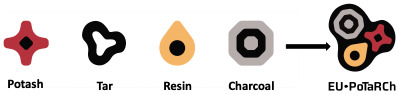
Symboles of potash, tar, resin, charcoal and logo of EU-PoTaRCh Action doi: http://dx.doi.org/10.18150/O75Y0C.

Potash, tar, resin, and charcoal (PoTaRCh) are four materials closely linked in their materiality, of extraordinary importance for societal resource use and the most significant products of non-timber forest use in Europe. ( Figure 1). The production of these materials is partly overlapping and intrinsically linked. All these products, including the extractives such as tannins, flavonoids, alkaloids, phenols etc, are potentially important resources in the context of renewable materials and bio-economy. Understanding the diverse production methods from the past, along with their positive and negative impacts on societies and environments across different regions of Europe since prehistory, is crucial. This knowledge will not only support the current preservation of (bio)cultural heritage but also inform future strategies for sustainable raw material supply.

To date, no comprehensive research has been conducted across the natural, social, applied, and humanities sciences on PoTaRCh to fully understand the scale and impact of its production on communities and societies, its significance for the natural environment, and its relevance to the current economy on a continental or even global scale. Consequently, there has been a grassroots need among scientists, tradition bearers, museum professionals, producers, and association representatives, to initiate a COST Action to address this challenge. In this article, we are pleased to celebrate and introduce the COST Action EU-PoTaRCh ^1^.

## COST inclusiveness policies in EU-PoTaRCh

**Figure 2.**
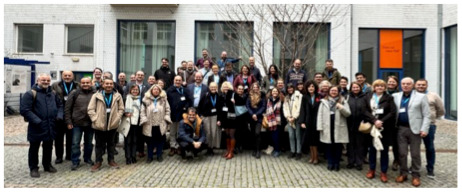
1 ^st^ General Meeting of Network for forest by-products charcoal, resin, tar, potash (COST Action EU-PoTaRCh), 5–7 March, 2024, Academy of Sciences of the Czech Republic, Prague Czech Republic.

Steps towards successful COST inclusiveness policies have already been implemented. To date, EU-PoTaRCh includes partners from 30 COST member countries, including 17 Inclusion Target Countries (ITCs), as well as partners from Mexico, Morocco and South Africa. ITC representatives constitute 57% of our members. The Action has achieved gender balance, with a similar participation rate of men and women, despite the topic traditionally being male-dominated. We are also actively working to attract young researchers, whose participation is a priority for EU-PoTaRCh, as the initiative is primarily addressed to them ( Figure 1). Our success is evident in the fact that our network includes 163 academic institutions in Europe and around the world, which guarantees broad, deep and multi-faceted PoTaRCh research. ( Figure 2).

## Building the capacity of EU-PoTaRCh

EU-PoTaRCh sets itself ambitious tasks aimed at expanding the network and building the potential of the Action ^2^:

- Mentoring and promotion of young people, especially from ITCs, where there are many active tradition bearers. Young people can easily combine research with practice.
- Creating an interdisciplinary collaborative network brings together excellence in history, archaeology, natural sciences and technology across Europe to support joint research on forest by-products to expand and exchange knowledge and experiences.
- Establishing a network on the future prospects of PoTaRCh for the bioeconomy and forest transformation. The White Paper for policy-makers and international organizations will be an important outcomesupporting the innovation potential of European society.
- Creating opportunities for interdisciplinary cooperation between scientists, practitioners (tradition bearers) museums, and enterprises in the field of bioeconomy, forest transformation and tourism.
- Facilitating the creation of a network of museums, associations of active tradition bearers, communities and other stakeholders, leading to the preparation of a European plan for the protection of the tangible and intangible heritage of PoTaRCh.
- Disseminating knowledge and experiences resulting from EU PoTaRCh activities among the general public through publications (reports and articles), workshops, conferences, an easily accessible, user-friendly website, social media and other media appearances.

The above objectives are pursued through various activities organized within the Action:

- We arrange meetings, workshops, and conferences that cater to all Action members, focusing closely on EU PoTaRCh deliverables. These activities may target specific groups (e.g., individual working groups) or involve all Action members.
- Short-Term Scientific Missions (STSM) grants, Inclusion Target Countries Conference (ITC) grants and Dissamination Conference (DC) grants are available for all Action members to apply for. These initiatives aim to foster international collaboration and knowledge exchange.
- Training schools provide intensive education on EU-PoTaRCh at host institutions' headquarters. These schools primarily benefit young researchers from across Europe, although participation is open to all interested individuals.
- We also organize webinars where every EU PoTaRCh member can present their ideas, research, experiences, projects, or opinions.
- We initiate, help in organizing and finding partners for joint research, scientific projects, publications, popularization and promotional activities or dissemination of knowledge about PoTaRCh. Additionally, we provide financial support for open access publications..

All these efforts promote cooperation, networking, knowledge sharing, and research and development within the framework of the EU PoTaRCh Action.

## Description of the Working Groups in EU-PoTaRCh

**Figure 3.**
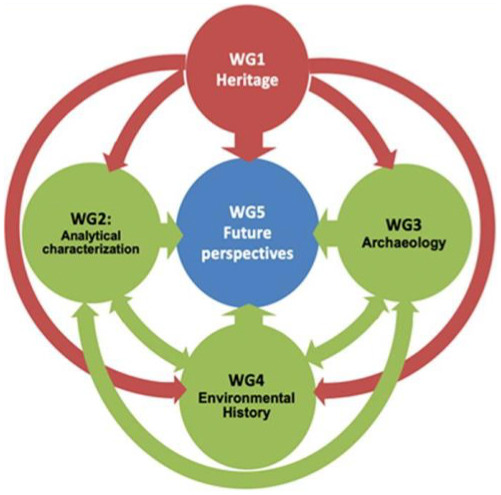
Network of Working Groups connections UE-PoTaRCh doi: http://dx.doi.org/10.18150/O75Y0C.

All objectives of the EU-PoTaRCh Action are implemented within the activities of five Working Groups (WGs) each focusing on different approaches to the past, present, and future of PoTaRCh, all strongly interconnected and unified under the heritage aspect ^2^. WG 1 compiles detailed definitions of traditional skills, knowledge, and technologies, along with their current applications. WGs 2, 3, and 4 represent various scientific approaches: laboratory analytics, archaeology, and environmental history, respectively. Finally, WG 5 evaluates future perspectives. ( Figure 3).

The production processes of PoTaRCh materials show both similarities and differences that have not yet been fully identified and described at European level, includingtheir areas of application. These processes can be traced thanks to archaeological research and historical written sources. Their survival is primarily ensured by local producers (especially in ITC and outside Europe), non-governmental organizations and associations dealing with the protection of intangible cultural heritage. Therefore objectives of WG1 (HERITAGE) are:

- Identification of cultural heritage related to PoTaRCh,
- Develop and implement measures to protect both tangible (historical places of production) and intangible (traditional knowledge) cultural heritage related to PoTaRCh at European level,
- Promoting the PoTaRCh heritage and ensuring its preservation for the future, while strengthening the social and economic position of the tradition bearers,
- Identification of PoTaRCh-related measures and policies that can benefit the tourism industry as well as local and regional development.

The characterization of PoTaRCh materials enables the identification of their chemical and structural composition, monitoring of compositional changes over time, detection of decomposition products, and analysis of interactions with environmental factors such as soil. This detailed understanding offers extensive opportunities to determine the sources and types of raw materials for PoTaRCh production, production conditions and methods, and storage and usage methods. Consequently, this knowledge allows for a comprehensive interpretation of PoTaRCh's impact on communities, the economy, and the environment on both regional and global scales. Therefore WG2 (ANALYTICAL CHARACTERIZATION) works with:

- Exploration the origins and traditional technologies by compositional analysis of PoTaRCh materials,
- Exploration of chemical/analytical approaches to characterize PoTaRCh materials based on analytical and archaeological chemistry as well as both organic and inorganic material characterization,
- Developing methodological approaches (including methods able to provide information on the biological origin of natural materials, technological and possible anthropogenic modifications) and analytical protocols as well as defining new research questions based on literature review, current production and experiments.

The production of charcoal and tar has left significant traces in the soil across many European cultural landscapes, while the production of potash is more challenging to trace archaeologically and often survives only in field names. Despite significant research identifying and assessing historical production sites, there is a lack of coordinated research at the European level. Therefore, WG3 (ARCHAEOLOGY) focuses on:

- Identifying the archaeological remnants of PoTaRCh production sites in the soil ("soil monuments"),
- Designing and implementing a standardized methodological approach for data exploration, validation, and characterization across Europe,
- Identifying and documenting the presence and absence of PoTaRCh sites and complexes in European cultural landscapes, while also understanding the contributing factors.

The environmental history of PoTaRCh, including the relationship between the environment, society, sustainable development and the socio-ecological system along with related changes from the local to the global level, remains largely unknown. The production technology of PoTaRCh is influenced, among other factors, by natural conditions. PoTaRCh production technology has not yet been systematically compared on a local to European scale within the context of the natural environment. Therefore, WG4 (ENVIRONMENTAL HISTORY) has the following task:

- Reconstruct and investigate the short- and long-term consequences of PoTaRCh production and use on socio-ecological systems in Europe and beyond,
- Identify human and non-human actors, examine knowledge transfer among producers and across different fields, investigate transportation and mobility aspects, and political and economic dimensions to promote sustainable management of natural resources,
- Compare PoTaRCh production technologies on various scales, from local to European.

COST Action EU-PoTaRCh asks the question: how can PoTaRCh products contribute to global challenges related to reducing reliance on fossil carbon sources? There are a few renewable sources of chemicals that can effectively compete with fossil sources, and PoTaRCh products represent a potentially promisingalternative. WG5 (FUTURE PERSPECTIVE) explores how trade in forest by-products can meet global challenges, facilitated bythe following tasks:

- Assessing the future economic prospects of the PoTaRCh heritage,
- Defining current and potential future products and methods relevant to the bioeconomy,
- Highlighting potential threats and challenges stemming from tradition and history

## Conclusions

We anticipate that in the coming years, the COST EU PoTaRCh Action will represent a significant milestone in the extensive history of PoTaRCh products. However, realizing this goal hinges entirely on the dedication and active involvement of current and prospective Action members. We hope that this article has inspired you to consider joining us in fostering a broader and more resilient network for PoTaRCh.

## Data Availability

No data are associated with this article.

## References

[1] For more information about COST. Reference Source

[2] For more information about COST Action CA22155. Reference Source

