# Network for forest by-products charcoal, resin, tar, potash (COST Action EU-PoTaRCh)

**DOI:** 10.12688/openreseurope.18160.2

**Published:** 2024-10-17

**Authors:** Magdalena Zborowska, Jakub Brózdowski, Jakob Starlander, Jiri Woitsch, Erika Ribechini, Rodica-Mariana Ion, Oliver Nelle, Koen Deforce, Anna Varga, Péter Szabó, Elena Badea, Johannes Tintiner-Olifiers, Katja Tikka, Jeannette Jacqueline Lucejko

**Affiliations:** 1 Department of Chemical Wood Technology, Faculty of Forestry and Wood Technology, Poznań University of Life Sciences, Wojska Polskiego, Poznań, 60-637, Poland; 2 Universität Bern, Länggassstrasse 49, Bern, 3012, Switzerland; 3 Institute of Ethnology of the Czech Academy of Sciences, Na Florenci 3, Praha, 11000, Czech Republic; 4 Department of Chemistry and Industrial Chemistry, University of Pisa, Pisa, 56124, Italy; 5 National Institute of Research and Development for Chemistry and Petrochemistry - ICECHIM, Bucharest, 060021, Romania; 6 University Valahia from Targoviste, 13 Aleea Sinaia, Targoviste, Dambovita, 130004, Romania; 7 Baden-Württemberg State Office for Cultural Heritage, Fischersteig 9, Gaienhofen-Hemmenhofen, 78343, Germany; 8 Ghent University, Sint-Pietersnieuwstraat 35, Gent, 9000, Belgium; 9 Belgium & Royal Belgian Institute of Natural Sciences, Vautierstraat 29, Brussels, 1000, Belgium; 10 Padon Foundation, Krúdy Gyula utca 16-18, Budapest, 1088, Hungary; 11 Institute of Botany of the Czech Academy of Sciences, Pruhonice, 25243, Czech Republic; 12 Department of Environmental Studies, Faculty of Social Studies, Masaryk University, Joštova 10, Brno, 60200, Czech Republic; 13 University of Craiova, Calea Bucuresti 187, Craiova, 200585, Romania; 14 EY Denkstatt – EY denkstatt GmbH, Wien, 1140, Austria; 15 University of Helsinki, Fabianinkatu 33, Helsinki, 00014, Finland

**Keywords:** Cultural heritage, history, archaeology, forest by-products, bio-economy, chemical compostion, physical properties

## Abstract

The COST EU-PoTaRCh Action establishes a network focused on the past, present, and future significance, production, and use of major forest by-products in Europe and beyond. The Action centers around forest by-products—primarily potash, tar, resin, and charcoal (PoTaRCh), along with plant extracts—which have been produced and utilized for over 100,000 years due to their unique chemical, biological, and therapeutic properties.

The primary goal of the Action is to demonstrate the importance of these products for the socio-economic development of European countries and beyond, as well as their impact on biodiversity and the natural environment. The Action's objectives are organized into five Working Groups (WGs), each aligned with specific areas of interest: heritage, chemical characterization, archaeology, environmental history, and future perspectives of PoTaRCh materials.

A key aspect of the Action is its support for stakeholders outside the scientific community who possess knowledge of PoTaRCh products through their use in industries such as production, education, and the promotion of forests' natural and cultural heritage. In doing so, the Action brings together stakeholders with diverse activity profiles, including museums, state forests, the forestry industry, associations dedicated to preserving traditions, and the tourism sector.

The EU-PoTaRCh Action adheres to the three key principles of COST’s inclusiveness policy: participation of inclusiveness target countries, gender balance, and the involvement of young researchers, including in leadership positions.

## Plain Language Summary

The COST EU-PoTaRCh Action was established to gather information on the past, present, and future significance, production, and use of major forest by-products in Europe and beyond. These products include potash, tar, resin, and charcoal (PoTaRCh), as well as plant extracts. They have played a crucial role on both a micro scale, affecting the daily lives of individuals, and a macro scale, influencing socio-economic parameters and the natural environment.

Given the extensive scope and historical depth of PoTaRCh production and use (with tar production dating back 100,000 years), knowledge on this subject across Europe and beyond is dispersed and highly diverse. The aim of the Action is to consolidate this information and enhance awareness of the importance of PoTaRCh products.

A key aspect of the Action is supporting stakeholders who possess knowledge of PoTaRCh products and are interested in their application in production, education, and the promotion of forest natural and cultural heritage. The Action brings together partners with varied activity profiles, including museums, state forests, the forest industry, associations dedicated to preserving traditions, the tourism industry, and the scientific community.

EU-PoTaRCh adheres to three key principles of COST’s inclusiveness policy: participation of Inclusiveness Target Countries (ITCs), gender balance, and the involvement of young researchers (YRs), including in leadership positions.

## Thematic scope of the COST Action EU-PoTaRCh

The COST Action EU-PoTaRCh creates a dynamic network focused on the enduring and evolving uses of Europe’s key forest by-products: potash, tar, resin, and charcoal (PoTaRCh). Rooted in traditional forestry practices, these natural resources are valued for their unique chemical, biological, and therapeutic properties.

The scientific mission of this initiative is to demonstrate how these by-products have historically driven community development, boosted economic vitality, and influenced biodiversity and climate. By tracing their use from past to present, EU-PoTaRCh seeks to understand the social and environmental roles of PoTaRCh materials, promote sustainable economic development, and draw valuable lessons for the future.

A core aspect of EU-PoTaRCh is its support for stakeholders passionate about PoTaRCh products and their cultural heritage. These stakeholders include experts involved in production, education, and promotion. The Action unites a diverse array of partners—museums, state forests, the forestry industry, tradition bearers, and the tourism sector—to address various needs and foster collaboration.

The initiative also emphasizes inclusivity by engaging countries with a rich history of PoTaRCh development, ensuring gender balance, and promoting young researchers into leadership roles. By adhering to these principles, EU-PoTaRCh not only preserves the legacy of forest by-products but also promotes innovation and sustainability, enhancing cultural and economic resilience across Europe.

**Figure 1.**
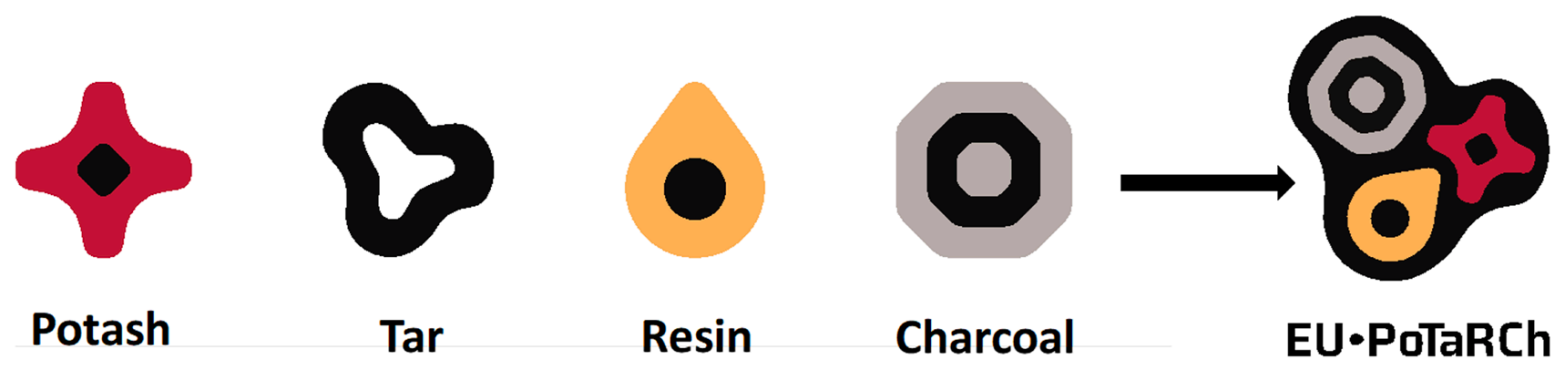
Symbols of potash, tar, resin, charcoal and logo of EU-PoTaRCh Action doi: 10.18150/O75Y0C.

Potash, tar, resin, and charcoal (PoTaRCh) are four materials closely connected in their production and of extraordinary importance for societal resource use, representing the most significant non-timber forest products in Europe (Figure 1). Their production methods overlap and are intrinsically linked. All of these products, including extractives such as tannins, flavonoids, alkaloids, and phenols, are potentially critical resources in the context of renewable materials and the bio-economy. Understanding the diverse production methods from the past, along with their positive and negative impacts on societies and environments across various regions of Europe since prehistory, is essential. This knowledge will not only support the current preservation of (bio)cultural heritage but also inform future strategies for sustainable raw material supply.

To date, no comprehensive research has been conducted across the natural, social, applied, and humanities sciences to fully understand the scale and impact of PoTaRCh production on communities and societies, its significance for the natural environment, or its relevance to the modern economy, both at the continental and global level. As a result, there has been a grassroots initiative from scientists, tradition bearers, museum professionals, producers, and association representatives to launch a COST Action aimed at addressing this challenge. In this article, we are pleased to introduce the COST Action EU-PoTaRCh.

## COST inclusiveness policies in EU-PoTaRCh

**Figure 2.**
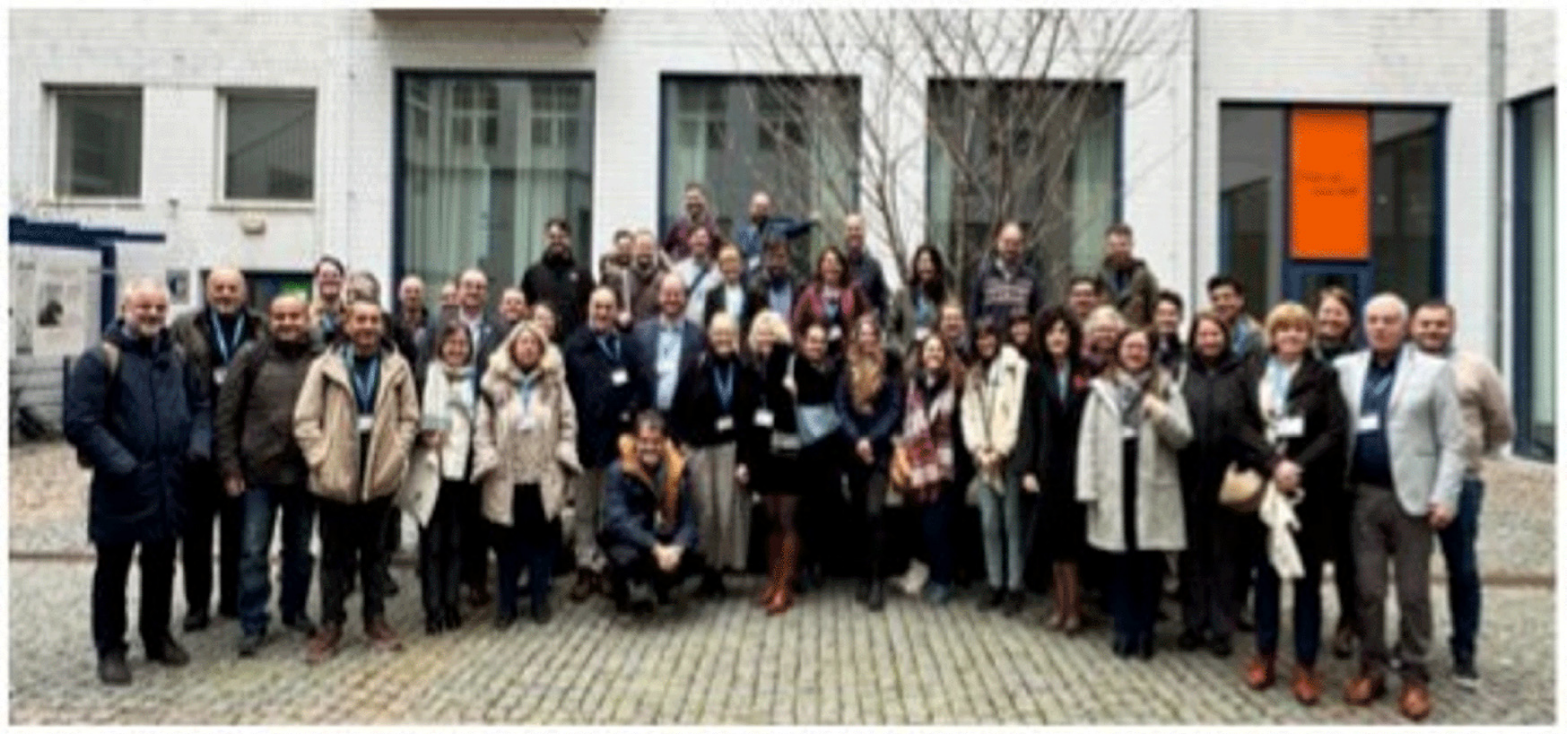
1 ^st^ General Meeting of Network for forest by-products charcoal, resin, tar, potash (COST Action EU-PoTaRCh), 5–7 March, 2024, Academy of Sciences of the Czech Republic, Prague Czech Republic.

Steps towards successful COST inclusiveness policies have already been implemented. To date, EU-PoTaRCh includes partners from 30 COST member countries, including 17 Inclusion Target Countries (ITCs), as well as partners from Mexico, Morocco and South Africa. ITC representatives constitute 57% of our members. The Action has achieved gender balance, with a similar participation rate of men and women, despite the topic traditionally being male-dominated. We are also actively working to attract young researchers, whose participation is a priority for EU-PoTaRCh, as the initiative is primarily addressed to them ( Figure 1). Our success is evident in the fact that our network includes 163 academic institutions in Europe and around the world, which guarantees broad, deep and multi-faceted PoTaRCh research. ( Figure 2) ^1^.

## Building the capacity of EU-PoTaRCh

EU-PoTaRCh sets itself ambitious tasks aimed at expanding the network and building the potential of the Action ^2^:

- Mentoring and promotion of young researchers, particularly from Inclusiveness Target Countries (ITCs), where many active tradition bearers reside. Young researchers have the opportunity to seamlessly combine research with practical applications. 

- Creating an interdisciplinary collaborative network that unites excellence in history, archaeology, natural sciences, and technology across Europe, fostering joint research on forest by-products to broaden and exchange knowledge and expertise.

- Establishing a network focused on the future prospects of PoTaRCh within the context of the bioeconomy and forest transformation. A significant outcome of this effort will be the publication of a White Paper for policymakers and international organizations, aimed at unlocking the innovation potential of European society.

- Providing opportunities for interdisciplinary cooperation among scientists, practitioners (tradition bearers), museums, and enterprises involved in the bioeconomy, forest transformation, and tourism. This collaboration will lead to the development of a European plan for the protection of both tangible and intangible PoTaRCh heritage.

- Disseminating knowledge and experiences gained through EU-PoTaRCh activities to the wider public via publications (reports and articles), workshops, conferences, an accessible and user-friendly website, social media, and other media channels.

The above objectives are pursued through various activities organized within the Action:

- **Meetings, workshops, and conferences** are arranged for all Action members, with a focus on key EU-PoTaRCh deliverables. These events may be tailored for specific groups (e.g., individual working groups) or open to all members.

**- Short-Term Scientific Missions (STSM) grants, Inclusion Target Countries (ITC) Conference grants, and Dissemination Conference (DC) grants** are available for all Action members. These initiatives aim to promote international collaboration and knowledge sharing.

- **Training schools** provide intensive courses on EU-PoTaRCh topics, hosted by participating institutions. These schools primarily benefit young researchers from across Europe but are open to all interested participants.

- **Webinars** are organized, offering EU-PoTaRCh members the opportunity to present their ideas, research, experiences, projects, or viewpoints.

- **We initiate and assist in organizing joint research projects, finding partners for scientific projects, publications,** outreach, and promotional activities related to PoTaRCh. Additionally, we offer **financial support** for open access publications.

All these efforts promote cooperation, networking, knowledge sharing, and research and development within the framework of the EU PoTaRCh Action.

## Description of the Working Groups in EU-PoTaRCh

**Figure 3.**
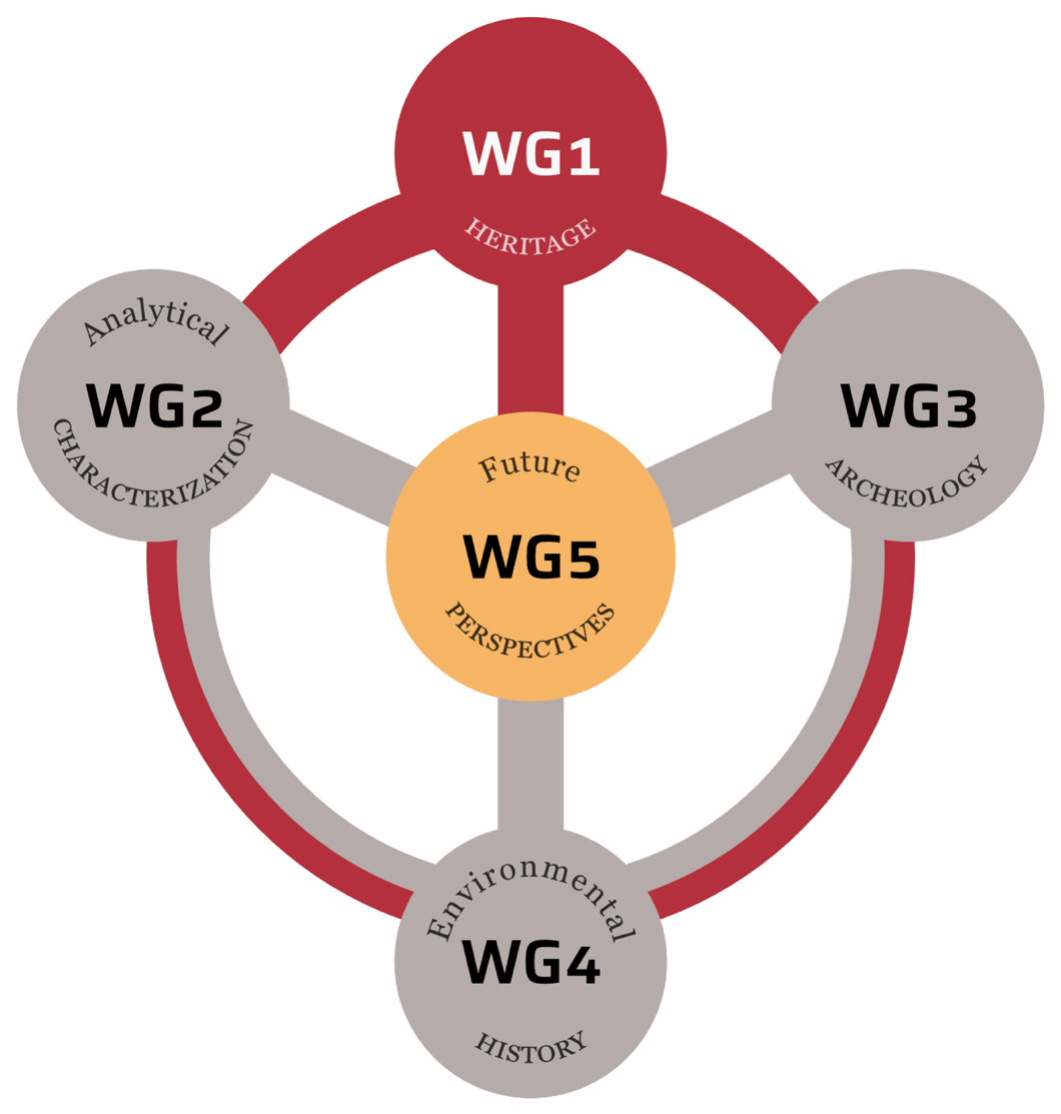
Network of Working Groups connections UE-PoTaRCh doi: 10.18150/O75Y0C.

All objectives of the EU-PoTaRCh Action are pursued through the activities of five Working Groups (WGs) each focusing on different approaches to the past, present, and future of PoTaRCh, all strongly interconnected and unified under the heritage aspect ^2^. WG 1 compiles detailed definitions of traditional skills, knowledge, and technologies, along with their current applications. WGs 2, 3, and 4 represent various scientific approaches: laboratory analytics, archaeology, and environmental history, respectively. Finally, WG 5 evaluates future perspectives. ( Figure 3).

The production processes of PoTaRCh materials show both similarities and differences that have not yet been fully identified and described at European level, includingtheir areas of application. These processes can be traced thanks to archaeological research and historical written sources. Their survival is primarily ensured by local producers (especially in ITC and outside Europe), non-governmental organizations and associations dealing with the protection of intangible cultural heritage. Therefore objectives of WG1 (HERITAGE) are:

- Identifying the cultural heritage related to PoTaRCh,
- Developing and implementing the measures to protect both tangible (historical places of production) and intangible (traditional knowledge) cultural heritage related to PoTaRCh at European level,
- Promoting the PoTaRCh heritage and ensuring its preservation for the future, while strengthening the social and economic position of the tradition bearers,
- Identifying the measures and policies related to PoTaRCh that can benefit the tourism industry as well as local and regional development.

The characterization of PoTaRCh materials enables the identification of their chemical and structural composition, monitoring of compositional changes over time, detection of decomposition products, and analysis of interactions with environmental factors such as soil. This detailed understanding offers extensive opportunities to determine the sources and types of raw materials for PoTaRCh production, production conditions and methods, and storage and usage methods. Consequently, this knowledge allows for a comprehensive interpretation of PoTaRCh’s impact on communities, the economy, and the environment on both regional and global scales. Therefore, WG2 (ANALYTICAL CHARACTERIZATION) works with:

- Exploring the origins and traditional technologies through compositional analysis of PoTaRCh materials.
- Developing chemical and analytical approaches to characterize PoTaRCh materials, utilizing techniques from analytical and archaeological chemistry, and encompassing both organic and inorganic material characterization.

The production of charcoal and tar has left significant traces in the soil across many European cultural landscapes, while the production of potash is more challenging to trace archaeologically and often survives only in field names. Despite significant research identifying and assessing historical production sites, there is a lack of coordinated research at the European level. Therefore, WG3 (ARCHAEOLOGY) focuses on:

- Identifying the archaeological remnants of PoTaRCh production sites in the soil ("soil monuments"),
- Designing and implementing a standardized methodological approach for data exploration, validation, and characterization across Europe,
- Identifying and documenting the presence and absence of PoTaRCh sites and complexes in European cultural landscapes, while also understanding the contributing factors.

The environmental history of PoTaRCh, including the relationship between the environment, society, sustainable development and the socio-ecological system along with related changes from the local to the global level, remains largely unknown. The production technology of PoTaRCh is influenced, among other factors, by natural conditions. PoTaRCh production technology has not yet been systematically compared on a local to European scale within the context of the natural environment. Therefore, WG4 (ENVIRONMENTAL HISTORY) has the following task:

- Reconstructing and investigating the short- and long-term consequences of PoTaRCh production and use on socio- ecological systems in Europe and beyond,
- Identifying human and non-human actors, examine knowledge transfer among producers and across different fields, investigate transportation and mobility aspects, and political and economic dimensions to promote sustainable management of natural resources,
- Comparing PoTaRCh production technologies on various scales, from local to European.

COST Action EU-PoTaRCh asks the question: how can PoTaRCh products contribute to global challenges related to reducing reliance on fossil carbon sources? There are a few renewable sources of chemicals that can effectively compete with fossil sources, and PoTaRCh products represent a potentially promisingalternative. WG5 (FUTURE PERSPECTIVE) explores how trade in forest by-products can meet global challenges, facilitated bythe following tasks:

- Assessing the future economic prospects of the PoTaRCh heritage,
- Defining current and potential future products and methods relevant to the bioeconomy,
- Highlighting potential threats and challenges stemming from tradition and history

## Conclusions

We anticipate that in the coming years, the COST EU PoTaRCh Action will represent a significant milestone in the extensive history of PoTaRCh products. However, realizing this goal hinges entirely on the dedication and active involvement of current and prospective Action members. We hope that this article has inspired you to consider joining us in fostering a broader and more resilient network for PoTaRCh.

## Data Availability

No data are associated with this article.

## References

[1] For more information about COST. Reference Source

[2] For more information about COST Action CA22155. Reference Source

