# Supplementary material for: Network for forest by-products charcoal, resin, tar, potash (COST Action EU-PoTaRCh)
Source: Open Res Eur. 2024 Oct 17;4:176. Originally published 2024 Aug 13. [Version 2] doi: 10.12688/openreseurope.18160.2 (PMC11489833; doi:10.12688/openreseurope.18160.2)
Supplement: Peer review history

# Reviewer report: Reviewer response for version 2

**DOI:** 10.21956/openreseurope.20122.r47915

**Published:** 2025-01-08

**Authors:** Dietrich Darr, Kathrin Meinhold

**Affiliations:** 1 Faculty of Agriculture, Food and Nutrition, Weihenstephan-Triesdorf University of Applied Sciences, Weidenbach, Germany

The open letter presents the rationale, organization and concrete research objectives of the COST Action EU-PoTaRCh in succinct and engaging way. The following minor observations may help to even further improve the document:

 1.) In addition to potash, tar, resin and charcoal, the abstract also lists plant extracts as a group of products that the Action will focus on. However, compared to the other (more conventional) products, this latter group is presented in less detail/ less prominently in the letter. For example, the various plant extracts are not mentioned before the third sentence of the 5th paragraph of the"thematic scope" section, giving the reader the impression that the Action will primarily focus on the other four products. Given the potential of plant extracts for the bioeconomy, this group of products could be presented more prominently in the letter. 

 2.) Potash, tar, resin, and charcoal are labelled he most significant non-timber forest products in Europe. This statement may be slightly exaggerated, at least for some regions, given the high regional relevance of NTFPs such as cork, forest mushrooms or berries. A source should be provided to back-up this statement.

 3.) The resolution of Fig. 2 is very low and could be improved.

 4.) There are minor typos that could be corrected, such as "includingtheir" (below Fig. 3), "promisingalterantives" and "bytheir" (before Conclusions).

Where applicable, are recommendations and next steps explained clearly for others to follow? (Please consider whether others in the research community would be able to implement guidelines or recommendations and/or constructively engage in the debate)

Yes

Does the article adequately reference differing views and opinions?

Yes

Are all factual statements correct, and are statements and arguments made adequately supported by citations?

Partly

Is the rationale for the Open Letter provided in sufficient detail? (Please consider whether existing challenges in the field are outlined clearly and whether the purpose of the letter is explained)

Yes

Is the Open Letter written in accessible language? (Please consider whether all subject-specific terms, concepts and abbreviations are explained)

Yes

Reviewer Expertise:

Agricultural and NTFP value chains; diversification of smallholder agriculture; strategy and management of SMEs in the ag-food and forestry sector

We confirm that we have read this submission and believe that we have an appropriate level of expertise to confirm that it is of an acceptable scientific standard, however we have significant reservations, as outlined above.

# Reviewer report: Reviewer response for version 2

**DOI:** 10.21956/openreseurope.20122.r48360

**Published:** 2024-12-27

**Authors:** Piergiuseppe Morone, Asha Khosravi

**Affiliations:** 1 UnitelmaSapienza University of Rome, Rome, Italy; 2 Universita degli Studi di Roma Unitelma Sapienza, Roma, Lombardia, Italy

**Summary of the Article**

 The open letter outlines the objectives and activities of the COST Action EU-PoTaRCh, which focuses on the historical, cultural, and economic significance of forest by-products such as potash, tar, resin, and charcoal. It highlights the interdisciplinary approach of the initiative, its commitment to inclusivity, and its efforts to involve diverse stakeholders, including researchers, tradition bearers, and industry professionals. While the letter successfully conveys the mission of the Action, there are areas where greater detail and clarity would improve its accessibility. Areas requiring further detail include the rationale, engagement with differing perspectives, and actionable guidance for stakeholders.

**Specific Questions**

1. **Is the rationale for the Open Letter provided in sufficient detail?**
**Partly**
  - The rationale highlights the need for interdisciplinary research, however, it does not fully detail the challenges in the field (e.g., the reliance on GHG-intensive materials, fragmented research, or gaps in substitution knowledge), or explain how these gaps motivated the Action. The rationale could benefit from a more explicit discussion.

**Recommendation**:

1.
  - To strengthen the rationale, it would be beneficial to expand on systemic challenges (e.g., limited coordinated research across disciplines and market-level data gaps) that necessitate this Action; and to link these challenges directly to COST Action objectives, framing them within global sustainability frameworks like the SDGs or Global Forest Goals (GFGs).
2. **Does the article adequately reference differing views and opinions?**
**Partly**
  - The letter outlines its scope effectively but does not sufficiently engage with alternative perspectives or address potential risks of forest product expansion (e.g., deforestation or overharvesting) and possible trade-offs, which would provide a broader context for the initiative. 

**Recommendation**:

1.
  - It might be worth mentioning trade-offs, such as overharvesting risks, leakage effects, or resource competition.
  - Situate them within broader debates on bioeconomy strategies, referencing alternative approaches like biotechnology innovations or biodiversity-focused solutions.
2. **Are all factual statements correct, and are statements and arguments made adequately supported by citations?**
**Partly**
  - While the information appears accurate, key claims (e.g., about gender balance, the global impact of PoTaRCh, and the bio-economy potential) lack supporting examples or evidence.

**Recommendation**:

-
  - It is suggested to provide brief, illustrative examples to substantiate key points. Even brief examples or indirect citations would improve credibility. 

Please acknowledge uncertainties, such as variability in substitution effects or limited data on non-European applications.

1. **Is the Open Letter written in accessible language?**
**Yes**
  - The language is clear and appropriate for a diverse audience. However, terms such as “tradition bearers” and “global” should be briefly explained to avoid ambiguity. 

**Recommendations**:

1.
  - Briefly define "tradition bearers” to clarify its meaning for non-specialist readers. E.g. as “individuals or groups preserving and applying traditional knowledge and practices.”
  - Please specify whether “global” refers to trade networks, environmental implications, broader bioeconomy impact, or knowledge dissemination beyond Europe.
2. **Where applicable, are recommendations and next steps explained clearly for others to follow?**
**Partly**
  - The letter outlines objectives and activities well, however, it does not provide sufficient actionable recommendations or details on how others can engage constructively.

**Recommendation**:

-
  - Clarify how researchers, policymakers, and other stakeholders can implement or contribute to the objectives of the COST Action. The reach and engagement of stakeholders could improve by specifying actionable steps.

**Key Points to Address**

1. **Strengthen the Rationale**
  - Elaborate on the challenges in the field (e.g., lack of comprehensive research across disciplines) and how the Action addresses these gaps.
  - Mention or expand on global challenges, such as reliance on GHG-intensive materials, overexploitation of resources, and gaps in market-level substitution data. It is suggested to articulate systemic challenges (e.g., competition for raw materials, lack of substitution data) and explicitly connect them to the Action’s objectives.
  - Link the goals more explicitly to the SDGs and GFGs, and align the rationale with global frameworks.

 

1. **Engage with Differing Perspectives**
  - Acknowledge trade-offs (e.g., resource competition or biodiversity risks) to present a balanced narrative.
  - If possible, reference alternative bioeconomy strategies to situate the Action within broader debates.
2. **Substantiate Claims**
  - Provide supporting evidence or references for claims about gender balance, global impacts, and the bio-economy. Even brief examples or indirect citations would improve credibility. Include specific examples to illustrate substitution benefits, gender balance efforts, and PoTaRCh’s contributions to sustainability.
  - Highlight uncertainties or knowledge gaps where relevant to provide nuance.

 

1. **Improve Recommendations and Next Steps**
2. Provide actionable steps for different stakeholders.
3. **Refine Terminology and Scope**
  - Define ambiguous terms like “tradition bearers” and clarify the scope of “global” to improve accessibility

Where applicable, are recommendations and next steps explained clearly for others to follow? (Please consider whether others in the research community would be able to implement guidelines or recommendations and/or constructively engage in the debate)

Partly

Does the article adequately reference differing views and opinions?

Partly

Are all factual statements correct, and are statements and arguments made adequately supported by citations?

Partly

Is the rationale for the Open Letter provided in sufficient detail? (Please consider whether existing challenges in the field are outlined clearly and whether the purpose of the letter is explained)

Partly

Is the Open Letter written in accessible language? (Please consider whether all subject-specific terms, concepts and abbreviations are explained)

Yes

Reviewer Expertise:

Expertise in sustainability transitions, circular bioeconomy, behavioural economics, and market assessments. Global health, public health and environmental health, human development, food security, and sustainability.

We confirm that we have read this submission and believe that we have an appropriate level of expertise to confirm that it is of an acceptable scientific standard, however we have significant reservations, as outlined above.

# Reviewer report: Reviewer response for version 2

**DOI:** 10.21956/openreseurope.20122.r45204

**Published:** 2024-10-18

**Authors:** Diego Tamburini

**Affiliations:** 1 The British Museum, London, England, UK

The authors have addressed most comments and I find the text improved. I have no further comment.

Where applicable, are recommendations and next steps explained clearly for others to follow? (Please consider whether others in the research community would be able to implement guidelines or recommendations and/or constructively engage in the debate)

Yes

Does the article adequately reference differing views and opinions?

No

Are all factual statements correct, and are statements and arguments made adequately supported by citations?

Partly

Is the rationale for the Open Letter provided in sufficient detail? (Please consider whether existing challenges in the field are outlined clearly and whether the purpose of the letter is explained)

Yes

Is the Open Letter written in accessible language? (Please consider whether all subject-specific terms, concepts and abbreviations are explained)

Yes

Reviewer Expertise:

I am an analytical chemist working on the characterisation and identification of organic materials in a cultural heritage context. I felt like I could assess all parts of the letter.

I confirm that I have read this submission and believe that I have an appropriate level of expertise to confirm that it is of an acceptable scientific standard.

# Reviewer report: Reviewer response for version 1

**DOI:** 10.21956/openreseurope.19629.r43523

**Published:** 2024-09-11

**Authors:** Anna Sandak

**Affiliations:** 1 University of Primorska, Glagoljaška, Koper, Slovenia; 2 InnoRenew CoE Livade, Izola, Livade, Slovenia

Dear authors, please find my comments below

 Abstract and Plain Language Summary provide the same information. Most of the sentences are repeated. Is there a reason for having both?

 You wrote: “ *COST Action EU-PoTaRCh weaves a dynamic network focused on the timeless and evolving uses of Europe’s key forest by-products: potash, tar, resin, and charcoal (PoTaRCh). These natural treasures, rooted in traditional forest practices, are celebrated for their unique chemical, biological, and therapeutic properties*”.

 I’m not sure if I understand correctly the author’s intention – what do you mean by the celebration of potash, tar, resin, and charcoal?

 Please standardize the grammatical style of the WG1 objectives. Currently, three different styles are being used: "identification…, develop…, promoting…". Choose one of these styles for consistency. The same applies to WG2, and it may be beneficial to maintain a uniform style across all WGs.

 Double check the paragraph that introduces WP5, some words are menged

 You stated that “ *EU-PoTaRCh not only preserves the legacy of forest by-products but also drives innovation and sustainability, enhancing cultural and economic resilience across Europe*”, which is very ambitious. This COST action has been active for almost one year but I’m sure you already faced some challenges. I’ve prepared a few questions that may guide further discussion and reflection related to four aspects:

 Identified Barriers and Mitigation Strategy: Have you encountered significant barriers that could impede progress toward the COST action objectives? If so, what strategies are in place to mitigate these challenges?

 Innovation and Sustainability: How specifically will this COST action drive the innovation and sustainability you mentioned? Are there particular areas or technologies where you see the most potential?

 Contribution to the Circular Bio-Economy: What is the planned approach to supporting the development of a circular bio-economy? How does EU-PoTaRCh intend to influence this transition, particularly through the use of forest by-products?

 Informing Future Strategies: How will this initiative contribute to the development of future strategies for sustainable raw material supply? Are there any key outputs or learnings that could shape future policy or industrial practices?

 I hope these questions will stimulate further discussion and refine the project’s direction as it continues to evolve.

Where applicable, are recommendations and next steps explained clearly for others to follow? (Please consider whether others in the research community would be able to implement guidelines or recommendations and/or constructively engage in the debate)

Partly

Does the article adequately reference differing views and opinions?

Yes

Are all factual statements correct, and are statements and arguments made adequately supported by citations?

Yes

Is the rationale for the Open Letter provided in sufficient detail? (Please consider whether existing challenges in the field are outlined clearly and whether the purpose of the letter is explained)

Partly

Is the Open Letter written in accessible language? (Please consider whether all subject-specific terms, concepts and abbreviations are explained)

Yes

Reviewer Expertise:

wood science and technology, material science

I confirm that I have read this submission and believe that I have an appropriate level of expertise to confirm that it is of an acceptable scientific standard.

# Reviewer report: Reviewer response for version 1

**DOI:** 10.21956/openreseurope.19629.r43528

**Published:** 2024-08-30

**Authors:** Diego Tamburini

**Affiliations:** 1 The British Museum, London, England, UK

The open-letter “Network for forest by-products charcoal, resin, tar, potash (COST Action EU-PoTaRCh) by Zborowska et al. presents the objectives and activities of this COST Action in a good way. The authors summarise the scope and relevance of the Action and promote the activities related to it in a convincing way. I find the letter mostly well-written and organised and I only have few suggestions to possibly improve its quality.

 The Abstract and Plain Language Summary only include the aims of the COST action, but not much on the plans to achieve these aims. These are described later, but probably mention this in the abstract would make it more complete.

 A few additional suggestions for the abstract/summary:

 Extractives could probably be replaced by “plant extractives” to make it clearer

 “along the time” does not sound the most appropriate here. Similarly, in the plain language summary, terms like “over the ages” are quite vague. The abstract also does not give a sense of the chronological scale that the research intends to cover. This should be clarified.

 Communities are mentioned, but which communities? This part can be better written. “their” also refers to different things, making the reading confusing.

 “impact on biodiversity and climate throughout time.” This is slightly obscure at this point. Also “throughout time” is not the most appropriate here.

 The authors distinguish between the “scientific objective” and the “general aim”. I found it slightly confusing. Why is one scientific and the other general?

 Terms like tradition/traditional are also used. I would encourage the authors to be careful about this or maybe try and find a way to define what they mean by tradition/traditional. It tends to get a sense of something that belongs to the past and has not changed, as opposed to modern “non-traditional” practices, but all this can be quite controversial and heritage institutions are trying to avoid this term. “Tradition bearers” is also used and I wonder if there is an alternative to this term.

 Some of the above comment also apply to the main text. This is generally clear and gives a better sense of the intentions and activities of the action. A few points follow.

 While I agree that “no comprehensive research has been conducted across the natural, social, applied, and humanities sciences…” some research is indeed available albeit not comprehensive. I would probably tone down this sentence. Generally, it would also be nice to mention an example of this topic being researched and conclusions being helpful to address the challenges presented, even if at a smaller scale of course.

 “despite the topic traditionally being male-dominated.” Is there evidence for this? It sounds slightly forces as it is presented now.

 “as the initiative is primarily addressed to them (Figure 1).” I don’t see how Figure 1 refers to young researchers. Maybe this is a typo?

 Some of the aims listed in “Building the capacity of EU-PoTaRCh” section feel slightly repetitive. For example, 2 and 4, but also some of the others. I wonder if these could be presented in a stronger way. Also, I think there is some confusion between objectives and activities. Some activities are presented in the objectives.

 Some language issues are present in the description of WG2, especially around the bullet points. The three bullet points also sound very similar to me. Could these be combined in a single point or maybe two?

 “Global” is often used throughout the article, but the focus being Europe, is global really appropriate in this context? European scale is sometimes mentioned and I found it more appropriate.

 The actions and activities of WG5 remain slightly vague. This part of the article could possibly be strengthened, maybe with an example? The sentence “There are a few renewable sources of chemicals that can effectively compete with fossil sources, and PoTaRCh products represent a potentially promising alternative.” could be expanded on. What few renewable sources of chemicals are the authors referring to? How are PoTaRCh products promising? Maybe referring to some already available research on this could be helpful to strengthen the argument.

 I think generally the letter could benefit from some bibliographical references putting it in context a bit more.

 The bullet points of WG5 are quite vague too.

 The conclusions could be strengthened too, maybe summarising the key messages of the Action.

Where applicable, are recommendations and next steps explained clearly for others to follow? (Please consider whether others in the research community would be able to implement guidelines or recommendations and/or constructively engage in the debate)

Yes

Does the article adequately reference differing views and opinions?

No

Are all factual statements correct, and are statements and arguments made adequately supported by citations?

Partly

Is the rationale for the Open Letter provided in sufficient detail? (Please consider whether existing challenges in the field are outlined clearly and whether the purpose of the letter is explained)

Yes

Is the Open Letter written in accessible language? (Please consider whether all subject-specific terms, concepts and abbreviations are explained)

Yes

Reviewer Expertise:

I am an analytical chemist working on the characterisation and identification of organic materials in a cultural heritage context. I felt like I could assess all parts of the letter.

I confirm that I have read this submission and believe that I have an appropriate level of expertise to confirm that it is of an acceptable scientific standard, however I have significant reservations, as outlined above.

# Response

**Published:** 2024-09-20

**Authors:** Magdalena Zborowska

**Affiliations:** Departament of Forestry nas Wood Technology, Poznań University of Life Science, Poznań, Poland

**Review Diego Tamburini** *Dear Reviewer,* *Thank you very much for your reviews of the article, I hope that the changes introduced will improve its value and make it more accessible to readers.* *On behalf of authors,* *Magdalena Zborowska * The open-letter “Network for forest by-products charcoal, resin, tar, potash (COST Action EU-PoTaRCh) by Zborowska et al. presents the objectives and activities of this COST Action in a good way. The authors summarise the scope and relevance of the Action and promote the activities related to it in a convincing way. I find the letter mostly well-written and organised and I only have few suggestions to possibly improve its quality.

**Comment: **The Abstract and Plain Language Summary only include the aims of the COST action, but not much on the plans to achieve these aims. These are described later, but probably mention this in the abstract would make it more complete. A few additional suggestions for the abstract/summary:

 Extractives could probably be replaced by “plant extractives” to make it clearer

 “along the time” does not sound the most appropriate here. Similarly, in the plain language summary, terms like “over the ages” are quite vague. The abstract also does not give a sense of the chronological scale that the research intends to cover. This should be clarified.

*Answer: Abstract has been improved according to suggestions.*

**Comment:** Communities are mentioned, but which communities? This part can be better written. “their” also refers to different things, making the reading confusing.

 “impact on biodiversity and climate throughout time.” This is slightly obscure at this point. Also “throughout time” is not the most appropriate here.

*Answer: Text has been improved according to suggestions.*

**Comment:** The authors distinguish between the “scientific objective” and the “general aim”. I found it slightly confusing. Why is one scientific and the other general?

*Answer: Text has been improved according to suggestions*

**Comment: **Terms like tradition/traditional are also used. I would encourage the authors to be careful about this or maybe try and find a way to define what they mean by tradition/traditional. It tends to get a sense of something that belongs to the past and has not changed, as opposed to modern “non-traditional” practices, but all this can be quite controversial and heritage institutions are trying to avoid this term.

*Answer: The term "traditional" is deeply ingrained in the subject matter of EU-PoTaRCh, and it is challenging to describe phenomena and actions without using it, even if it is not fully defined. However, the authors have removed the term where its inclusion was not essential and where its removal did not alter the meaning of the sentence.*

***Comment:*** *“Tradition bearers” is also used and I wonder if there is an alternative to* this term. *Answer: A more precise term might be "promoters/advocates/lovers of tradition." However, "tradition bearer" is a well-established term with universal recognition. Despite its awkwardness, we have retained it in certain places in the text where it is most appropriate.*

 Some of the above comment also apply to the main text. This is generally clear and gives a better sense of the intentions and activities of the action. A few points follow.

**Comment: **While I agree that “no comprehensive research has been conducted across the natural, social, applied, and humanities sciences…” some research is indeed available albeit not comprehensive. I would probably tone down this sentence. Generally, it would also be nice to mention an example of this topic being researched and conclusions being helpful to address the challenges presented, even if at a smaller scale of course.

*Answer: The purpose of the article was to outline the goals of the Action, describe our approach, and explain the rationale, without delving into the current state of knowledge. Given the broad scope of the Action, it is challenging to select literature from such diverse scientific areas without favoring a particular field, which we aimed to avoid in this overview article. As part of the Action, we plan to develop several review publications focused on different areas of interest as defined by the working groups. These reviews will be supported by a comprehensive range of literature sources.*  

**Comment:** “despite the topic traditionally being male-dominated.” Is there evidence for this? It sounds slightly forces as it is presented now.

 It is not forced. It is true that the Action, thanks to our efforts, manages to maintain gender balance. What is more, the scientific community is also represented by both sexes. One producer community, … is represented mainly by men.

*Answer: While the EU-PoTaRCh Action has made strides in maintaining gender balance within the scientific community, we recognize that the situation is different outside of it. Specifically, in sectors such as production and among tradition bearers and association members, there is a predominance of men. Given that one of the core principles of all COST Actions is to promote gender balance, we believe it is important to address this imbalance in non-scientific environments as well. Our articles aim to highlight our efforts to address this issue and accurately reflect the current gender disparities. Therefore, we will use the term that best describes the existing situation.*

**Comment:** “as the initiative is primarily addressed to them (Figure 1).” I don’t see how Figure 1 refers to young researchers. Maybe this is a typo?

*Answer: Of course it is mistake. We rejected this information in text.*  

**Comment: **Some of the aims listed in “Building the capacity of EU-PoTaRCh” section feel slightly repetitive. For example, 2 and 4, but also some of the others. I wonder if these could be presented in a stronger way.

*Answer: Thank you for your feedback. The points do indeed overlap and differ mainly in nuances. As a result, I combined points 4 and 5 without compromising the clarity of the text. Points 2 and 4 address different objectives: point 2 focuses on networking among scientific disciplines, while point 4 pertains to networking in non-scientific environments. Therefore, I have kept these two points separate.*  

**Comment: **Also, I think there is some confusion between objectives and activities. Some activities are presented in the objectives.

*Answer: Thank you for your observation. We appreciate your attention to the distinction between objectives and activities. While certain goals are indeed achieved through specific actions—such as using training schools and STSMs to mentor and promote young researchers, and organizing meetings, workshops, and conferences to foster interdisciplinary cooperation—this approach is central to the COST Action. For this reason, we prefer to keep the text as is. However, we will ensure that the distinction between objectives and activities is made clear where possible.*

**Comment: **Some language issues are present in the description of WG2, especially around the bullet points. The three bullet points also sound very similar to me. Could these be combined in a single point or maybe two?

*Answer: Thank you for your comment, in fact the last point contains the above, so it can be deleted.*

**Comment:** “Global” is often used throughout the article, but the focus being Europe, is global really appropriate in this context? European scale is sometimes mentioned and I found it more appropriate.

*Answer: You’re right that the term “global” can be challenging. While it is easier to focus on Europe, where most of our members are based, we cannot overlook the global nature of the issues related to PoTaRCh. Environmental changes, such as those affecting the climate due to PoTaRCh production, have global implications. Similarly, historical economic and social changes—such as PoTaRCh production in Europe and Africa and its subsequent trade in the United States—were global in scope. Despite having less detailed knowledge about other continents compared to Europe, we will strive to study PoTaRCh on a global scale because it is a topic with worldwide relevance.*

**Comment: **The actions and activities of WG5 remain slightly vague. This part of the article could possibly be strengthened, maybe with an example? The sentence “There are a few renewable sources of chemicals that can effectively compete with fossil sources, and PoTaRCh products represent a potentially promising alternative.” could be expanded on. What few renewable sources of chemicals are the authors referring to? How are PoTaRCh products promising? Maybe referring to some already available research on this could be helpful to strengthen the argument.

*Answer: Thank you for comment. As we mentioned previously, we try not to quote other articles in this article, it is only intended to provide general information about the campaign, encouraging you to look for more information on the website or in review articles.*

**Comment: **I think generally the letter could benefit from some bibliographical references putting it in context a bit more.

*Answer: We have already explained our idea.*

**Comment: **The bullet points of WG5 are quite vague too.

 The conclusions could be strengthened too, maybe summarising the key messages of the Action.

*Answer: Thank you for your valuable feedback. While we believe the bullet points in WG5 are specific and align with the objectives of the Action, we understand that they might seem somewhat unclear. We will review them to ensure they are more precise and effectively convey the intended details. Additionally, we will work on strengthening the conclusions by summarizing the key messages of the Action to provide a clearer and more impactful overview.*

**Is the rationale for the Open Letter provided in sufficient detail? (Please consider whether existing challenges in the field are outlined clearly and whether the purpose of the letter is explained)**

 Yes

**Does the article adequately reference differing views and opinions?** No **Are all factual statements correct, and are statements and arguments made adequately supported by citations?**

 Partly

**Is the Open Letter written in accessible language? (Please consider whether all subject-specific terms, concepts and abbreviations are explained)**

 Yes

**Where applicable, are recommendations and next steps explained clearly for others to follow? (Please consider whether others in the research community would be able to implement guidelines or recommendations and/or constructively engage in the debate)**
